# 2-(1,3-Dioxoisoindolin-2-yl)acetic acid–*N*′-[(*E*)-4-meth­oxy­benzyl­idene]pyridine-4-carbohydrazide (2/1)

**DOI:** 10.1107/S1600536812038032

**Published:** 2012-09-08

**Authors:** Sladjana B. Novaković, Goran A. Bogdanović, Shaaban K. Mohamed, Mustafa R. Albayati, Ayad S. Hameed

**Affiliations:** a ’Vinča’ Institute of Nuclear Sciences, Laboratory of Theoretical Physics and Condensed Matter Physics, University of Belgrade, PO Box 522, 11001 Belgrade, Serbia; b Manchester Metropolitan University, Chemistry and Environmental Division, Manchester M1 5GD, England; c Kirkuk University, College of Science, Department of Chemistry, Kirkuk, Iraq; d Chemistry Department, Tikrit University, Tikrit, Iraq

## Abstract

In the crystal structure of the title compound, 2C_10_H_7_NO_4_·C_14_H_13_N_3_O_2_, the two independent acid mol­ecules are connected through strong O—H⋯N and O—H⋯O hydrogen bonds to the central mol­ecule of the anti­tubercular drug *N*′-[(*E*)-4-meth­oxy­benzyl­idene]pyridine-4-carbohydrazide. Two such trimolecular units related by an inversion centre inter­act through a pair of N—H⋯O hydrogen bonds, forming a 3 + 3 mol­ecular aggregate. The dihedral angle between the aromatic rings of the hydrazone mol­ecule is 1.99 (12)°. The crystal packing features weak C—H⋯O and π–π stacking inter­actions, with centroid–centroid distances of 3.8460 (19) and 3.8703 (13) Å.

## Related literature

For anti-tuberculosis drugs containing the isoniazid core structure, see: Bijev (2006 ▶); Imramovský *et al.* (2007 ▶); Maccari *et al.* (2005 ▶); Schultheiss & Newman (2009 ▶); Shindikar & Viswanathan (2005 ▶); Sinha *et al.* (2005 ▶). For crystal structures with *N*′-[(*E*)-(4-meth­oxy­phen­yl)methyl­idene]pyridine-4-carbo­hydrazide, see: Jing *et al.* (2005 ▶); Lin & Liu (2007 ▶); Shanmuga Sundara Raj *et al.* (1999 ▶); Wardell *et al.* (2007 ▶). For crystal structures with 2-(1,3-dioxoisoindolin-2-yl)acetic acid, see: Barooah *et al.* (2006 ▶); Feeder & Jones (1994 ▶, 1996 ▶). For a related co-crystal, see: Mohamed *et al.* (2012 ▶). For the synthesis of 2-(1,3-dioxoisoindolin-2-yl)acetic acid, see: Rajpurohit & Sah (2005 ▶).
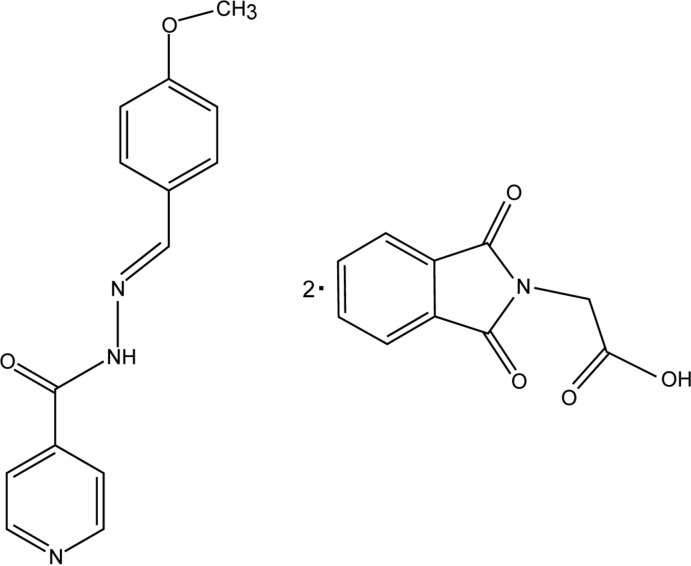


## Experimental

### 

#### Crystal data

2C_10_H_7_NO_4_·C_14_H_13_N_3_O_2_
*M* *_r_* = 665.61
Triclinic, 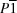

*a* = 8.1238 (4) Å
*b* = 12.7963 (7) Å
*c* = 15.9191 (11) Å
α = 105.590 (5)°
β = 101.160 (5)°
γ = 97.535 (4)°
*V* = 1534.19 (17) Å^3^
*Z* = 2
Cu *K*α radiation
μ = 0.91 mm^−1^
*T* = 293 K
0.16 × 0.10 × 0.08 mm

#### Data collection

Oxford Diffraction Xcalibur (Sapphire3, Gemini) diffractometer
Absorption correction: multi-scan (*CrysAlis PRO*; Oxford Diffraction, 2009 ▶) *T*_min_ = 0.963, *T*_max_ = 1.000
10334 measured reflections
5912 independent reflections
4836 reflections with *I* > 2σ(*I*)
*R*_int_ = 0.022

#### Refinement

*R*[*F* ^2^ > 2σ(*F* ^2^)] = 0.040
*wR*(*F* ^2^) = 0.116
*S* = 1.06
5912 reflections
456 parameters
H atoms treated by a mixture of independent and constrained refinement
Δρ_max_ = 0.28 e Å^−3^
Δρ_min_ = −0.21 e Å^−3^

### 

Data collection: *CrysAlis PRO* (Oxford Diffraction, 2009 ▶); cell refinement: *CrysAlis PRO*; data reduction: *CrysAlis PRO*; program(s) used to solve structure: *SHELXS97* (Sheldrick, 2008 ▶); program(s) used to refine structure: *SHELXL97* (Sheldrick, 2008 ▶); molecular graphics: *ORTEP-3* (Farrugia, 1997 ▶); software used to prepare material for publication: *WinGX* (Farrugia, 1999 ▶), *PLATON* (Spek, 2009 ▶) and *PARST* (Nardelli, 1995 ▶).

## Supplementary Material

Crystal structure: contains datablock(s) I, global. DOI: 10.1107/S1600536812038032/rz2799sup1.cif

Structure factors: contains datablock(s) I. DOI: 10.1107/S1600536812038032/rz2799Isup2.hkl

Supplementary material file. DOI: 10.1107/S1600536812038032/rz2799Isup3.cml

Additional supplementary materials:  crystallographic information; 3D view; checkCIF report

## Figures and Tables

**Table 1:** Hydrogen-bond geometry (Å, °)

*D*—H⋯*A*	*D*—H	H⋯*A*	*D*⋯*A*	*D*—H⋯*A*
O2*A*—H1*OA*⋯N1	1.00 (3)	1.60 (3)	2.5997 (19)	177 (2)
O2*B*—H1*OB*⋯O1	0.93 (2)	1.75 (2)	2.6736 (16)	170 (2)
N2—H1*N*2⋯O4*B* ^i^	0.87 (2)	2.22 (2)	3.0549 (18)	161 (2)
C2*A*—H2*A*2⋯O3*B* ^ii^	0.97	2.57	3.477 (2)	156
C5*B*—H5*B*⋯O1^iii^	0.93	2.51	3.158 (2)	126
C7*B*—H7*B*⋯O3*B* ^iv^	0.93	2.55	3.275 (2)	135
C5—H5⋯O3*A* ^v^	0.93	2.48	3.341 (2)	154

Symmetry codes: (i) 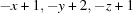; (ii) 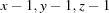; (iii) 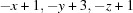; (iv) 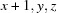; (v) 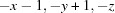.

**Fig. 1.**
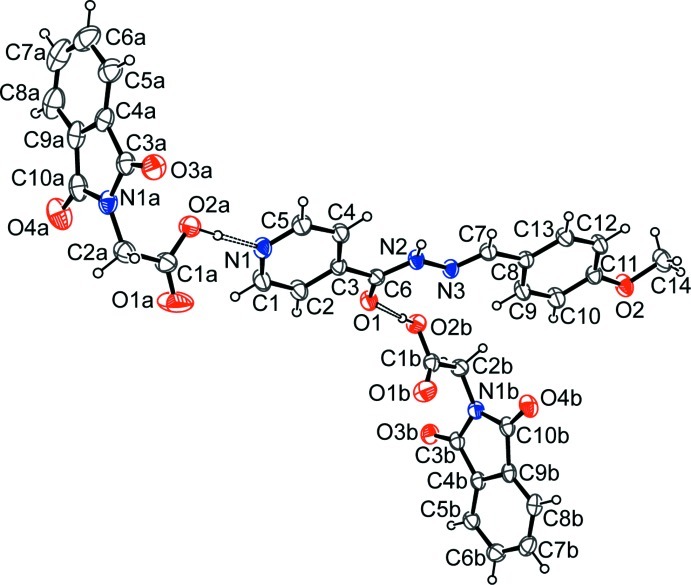
The molecular structure of the title compound with displacement ellipsoids drawn at the 35% probability level. Intermolecular hydrogen interactions are shown as dashed bonds.

**Fig. 2.**
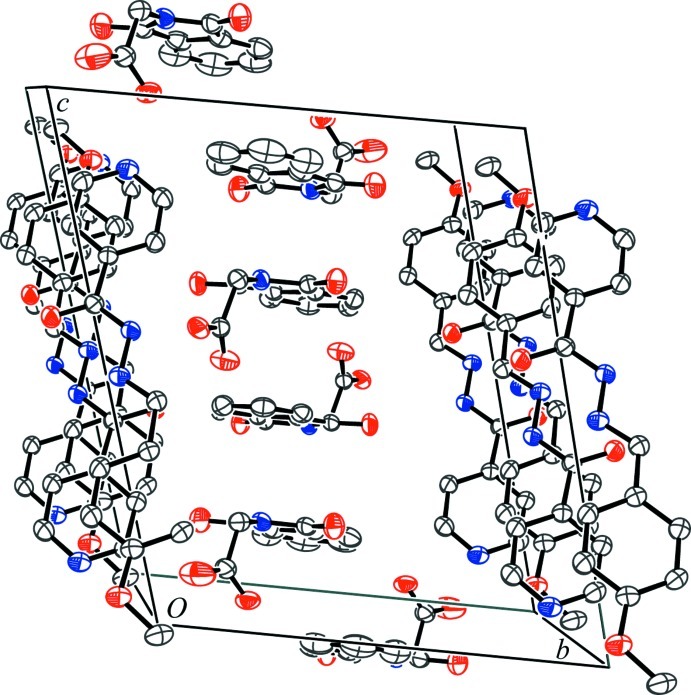
Crystal packing of the title compound, showing the stacking arrangement of the components within the unit cell.

## supplementary crystallographic information

### Comment

Compounds incorporating the isoniazid (INH) core structure have shown high inhibitory activity *in vitro* (Bijev, 2006; Imramovský *et al.*, 2007) and in mice towards *M*. tuberculosis H37Rv, ATCC 27294, *M*. tuberculosis clinical isolates and isoniazid-resistant *M*. tuberculosis (Maccari *et al.*, 2005; Schultheiss & Newman 2009; Shindikar & Viswanathan, 2005; Sinha *et al.*, 2005). In the present study we report the crystal structure of a novel, tricomponent cocrystal (I) containing the isoniazid-related hydrazone *N*'-[(*E*)-(4-methoxyphenyl)methylidene]pyridine-4-carbohydrazide and 2-(1,3-dioxoisoindolin-2-yl)acetic acid in a 1:2 molar ratio.

The asymmetric unit of (I) contains one hydrazone molecule and two crystallographically independent molecules of the acid denoted as A and B in Fig. 1. The A and B molecules tightly connect to the hydrazone *via* short and directional O2a—H1Oa···N1 and O2b— H1Ob···O1 hydrogen bond, respectively (Table 1). The interactions of A and B molecules significantly differ as their carboxyl acid groups, serving as proton donors, find different acceptors within the hydrazone molecule *i.e.* pyridinyl N1 and carboxyl O1 (Fig. 1). The interaction O2a—H1Oa···N1 which directly involves the acidic –COOH group and the most basic pyridinyl N1 atom causes the noticeable elongation of O2—H1Oa bond in molecule A (Table 1), yet no proton transfer occurs and all components remain neutral.

Besides the different engagement in the strongest interactions, the important difference between molecules A and B concerns their conformation. Thus the O1 carbonyl atom adopts *trans* and *cis* orientation relating to N1 atom in A and B. In addition, the O1—C1—C2—N1 torsion angle is 161.2 (2) and 1.4 (2)°, in molecules A and B respectively. It is worth mentioning that in a previously reported cocrystal (Mohamed *et al.*, 2012) as well as in the crystal structures of 2-(1,3-dioxoisoindolin-2-yl)acetic acid containing one molecule in the asymmetric unit (Feeder & Jones, 1996), two independent molecules (Barooah *et al.*, 2006) or the same molecule as monohydrate (Feeder & Jones, 1994), the value of the corresponding torsion angle O1—C1—C2—N1 is below 15.8° indicating the preferred conformation is similar to that of molecule B.

There are several crystal structures of hydrazone *N*'-[(*E*)-(4-methoxyphenyl)methylidene]pyridine-4-carbohydrazide describing this compound as monohydrate crystallizing in two forms, monoclinic (Jing *et al.*, 2005; Shanmuga Sundara Raj *et al.*, 1999; Wardell *et al.*, 2007) and orthorhombic (Lin & Liu, 2007). The present form of the molecule shows no particular difference in bond lengths and angles in comparison to the previous ones.

The above described trimer with strongly intermolecular hydrogen-bonded components (Fig. 1), undergoes further arrangement *via* much weaker interamolecular interactions. Two such trimolecular units related by an inversion centre interact through a pair of N2—H1N2···O4b hydrogen bonds (Table 1) forming a 3 + 3 molecular aggregate.

Apart from this classical N—H···O hydrogen bond, the arrangement of the molecules in the cocrystal is further based on weak C— H···O (Table 1) and π–π interactions. Figure 2 displays the three-dimensional crystal packing as viewed down the *a* axis. The molecules of hydrazone are stacked in the *ac* plane with the perpendicular interplanar distances of 3.44 [for molecule at (-*x*, -*y* + 2, -*z* + 1)] and 3.46 Å [for molecule at (-*x* + 1, -*y* + 2, -*z* + 1)]. On the other hand, the acid molecules arrange along the *c* axis in an AABBAABB sequence, with the perpendicular distances between the rings ranging from 3.31 to 3.43 Å. Considering only the six membered aromatic rings one can observe only a modest overlap: *Cg*1···*Cg*2 (-*x*, -*y* + 2, -*z* + 1) = 3.8460 (10) Å, where *Cg*1 and *Cg*2 are the centroids of the N1—C5 and C8—C13 rings, respectively and *Cg*4···*Cg*4 (*x* + 1/2, -*y* + 1, -*z*) 3.8703 (13) Å where *Cg*4 is the centroid of the C4a—C9a ring.

### Experimental

The cocrystallized solid (I) was obtained unintentionally from a reaction of 0.01 mol (2.55 g) of *N*'-[(E)-(4-methoxyphenyl)methylidene]pyridine-4-carbohydrazide with 0.02 mol (4.10 g) of (1,3-dioxo-1,3-dihydro-2*H*-isoindol-2-yl)acetic acid in 50 ml ethanol. The reaction mixture was heated for 6 h at 351 K, then poured on crushed ice (50 g). The resulting solid was filtered off, washed with cold ethanol and recrystallized from ethanol. Yellow crystals suitable for X-ray diffraction analysis were grown up in a diluted ethanolic solution over two days. M.p. 472- 474 K. Crystals of the title compound can also be obtained by a simple crystallization of two components dissolved in ethanol.

2-(1,3-Dioxoisoindolin-2-yl)acetic acid was prepared according to the literature procedure (Rajpurohit & Sah, 2005). *N*'-[(E)-(4-methoxyphenyl)methylidene]pyridine-4-carbohydrazide was prepared by the reaction of an equimolar solution of isoniazid (0.01 mol; 1.37 g) and *p*-methoxybenzaldehyde (0.01 mol; 1.21 g) in ethanol. The reaction mixture was heated at 351 K and monitored by TLC until completed after 4 h, then left at fume cupboard where the solvent evaporated. The resulting solid was recrystallized from ethanol in a very good yield (87%); m.p. 435–437 K.

### Refinement

H atoms bonded to C atoms were placed at calculated positions, with C—H distances fixed at 0.93 Å for aromatic C(*sp*^2^) atoms and at 0.96 and 0.97 Å for methyl and methylene C(*sp*^3^) atoms, respectively. The corresponding isotropic displacement parameters of the H atoms were set equal to 1.2*U*_eq_ or 1.5*U*_eq_ of the parent C(*sp*^2^) or C(*sp*^3^) atoms, respectively. A rotating model was employed for the methyl group. The H atoms attached to N and O atoms were located in a difference Fourier map and refined isotropically.

### Crystal data

**Table:**

2C_10_H_7_NO_4_·C_14_H_13_N_3_O_2_	*Z* = 2
*M**_r_* = 665.61	*F*(000) = 692
Triclinic, *P*1	*D*_x_ = 1.441 Mg m^−^^3^
Hall symbol: -P 1	Cu *K*α radiation, λ = 1.54184 Å
*a* = 8.1238 (4) Å	Cell parameters from 3871 reflections
*b* = 12.7963 (7) Å	θ = 3.0–72.3°
*c* = 15.9191 (11) Å	µ = 0.91 mm^−^^1^
α = 105.590 (5)°	*T* = 293 K
β = 101.160 (5)°	Prismatic, yellow
γ = 97.535 (4)°	0.16 × 0.10 × 0.08 mm
*V* = 1534.19 (17) Å^3^	

### Data collection

**Table:**

Oxford Diffraction Xcalibur (Sapphire3, Gemini) diffractometer	5912 independent reflections
Radiation source: Enhance (Cu) X-ray Source	4836 reflections with *I* > 2σ(*I*)
Graphite monochromator	*R*_int_ = 0.022
Detector resolution: 16.3280 pixels mm^-1^	θ_max_ = 72.5°, θ_min_ = 3.0°
ω scans	*h* = −6→9
Absorption correction: multi-scan (*CrysAlis PRO*; Oxford Diffraction, 2009)	*k* = −15→15
*T*_min_ = 0.963, *T*_max_ = 1.000	*l* = −19→19
10334 measured reflections	

### Refinement

**Table:**

Refinement on *F*^2^	Secondary atom site location: difference Fourier map
Least-squares matrix: full	Hydrogen site location: inferred from neighbouring sites
*R*[*F*^2^ > 2σ(*F*^2^)] = 0.040	H atoms treated by a mixture of independent and constrained refinement
*wR*(*F*^2^) = 0.116	*w* = 1/[σ^2^(*F*_o_^2^) + (0.0523*P*)^2^ + 0.272*P*] where *P* = (*F*_o_^2^ + 2*F*_c_^2^)/3
*S* = 1.06	(Δ/σ)_max_ = 0.001
5912 reflections	Δρ_max_ = 0.28 e Å^−^^3^
456 parameters	Δρ_min_ = −0.21 e Å^−^^3^
0 restraints	Extinction correction: *SHELXL97* (Sheldrick, 2008), Fc^*^=kFc[1+0.001xFc^2^λ^3^/sin(2θ)]^-1/4^
Primary atom site location: structure-invariant direct methods	Extinction coefficient: 0.0024 (3)

### Special details

**Table:**

Experimental. Absorption correction: Empirical absorption correction using spherical harmonics, implemented in SCALE3 ABSPACK scaling algorithm. 'CrysAlisPro, (Oxford Diffraction, 2009)'

### Fractional atomic coordinates and isotropic or equivalent isotropic displacement parameters (Å^2^)

**Table:**

	*x*	*y*	*z*	*U*_iso_*/*U*_eq_	
C1	−0.2642 (2)	0.95037 (14)	0.17010 (12)	0.0549 (4)	
H1	−0.3093	0.9940	0.1368	0.066*	
C2	−0.1425 (2)	1.00139 (13)	0.24952 (11)	0.0493 (4)	
H2	−0.1069	1.0778	0.2689	0.059*	
C3	−0.07405 (18)	0.93782 (11)	0.30007 (9)	0.0366 (3)	
C4	−0.1314 (2)	0.82441 (12)	0.26761 (11)	0.0451 (4)	
H4	−0.0881	0.7786	0.2992	0.054*	
C5	−0.2535 (2)	0.78039 (13)	0.18774 (11)	0.0508 (4)	
H5	−0.2915	0.7041	0.1667	0.061*	
C6	0.05450 (18)	0.99688 (11)	0.38761 (9)	0.0367 (3)	
C7	0.33689 (19)	0.92085 (12)	0.54388 (10)	0.0401 (3)	
H7	0.3112	0.8449	0.5156	0.048*	
C8	0.46634 (18)	0.96649 (12)	0.62817 (9)	0.0384 (3)	
C9	0.5239 (2)	1.08025 (13)	0.66447 (11)	0.0495 (4)	
H9	0.4785	1.1271	0.6346	0.059*	
C10	0.6462 (2)	1.12434 (13)	0.74335 (12)	0.0534 (4)	
H10	0.6852	1.2005	0.7656	0.064*	
C11	0.7124 (2)	1.05611 (13)	0.79041 (10)	0.0440 (3)	
C12	0.6576 (2)	0.94290 (13)	0.75570 (11)	0.0479 (4)	
H12	0.7014	0.8965	0.7865	0.057*	
C13	0.5365 (2)	0.89881 (13)	0.67442 (11)	0.0455 (4)	
H13	0.5017	0.8225	0.6505	0.055*	
C14	0.8949 (3)	1.04657 (17)	0.92371 (12)	0.0657 (5)	
H14A	0.8023	1.0151	0.9443	0.099*	
H14B	0.9823	1.0934	0.9744	0.099*	
H14C	0.9415	0.9884	0.8901	0.099*	
N1	−0.31974 (17)	0.84128 (11)	0.13922 (9)	0.0491 (3)	
N2	0.13864 (16)	0.93389 (10)	0.42826 (8)	0.0406 (3)	
N3	0.25843 (15)	0.98456 (10)	0.50851 (8)	0.0406 (3)	
O1	0.07630 (14)	1.09815 (8)	0.41843 (7)	0.0474 (3)	
O2	0.83335 (17)	1.11023 (10)	0.86773 (8)	0.0597 (3)	
C1A	−0.5342 (2)	0.78299 (14)	−0.07334 (12)	0.0535 (4)	
C2A	−0.6544 (2)	0.71923 (15)	−0.16347 (11)	0.0555 (4)	
H2A1	−0.6875	0.7711	−0.1952	0.067*	
H2A2	−0.5940	0.6709	−0.1989	0.067*	
C3A	−0.8216 (2)	0.54517 (13)	−0.15390 (10)	0.0475 (4)	
C4A	−0.9875 (2)	0.51700 (15)	−0.13116 (11)	0.0511 (4)	
C5A	−1.0643 (3)	0.42141 (17)	−0.11908 (12)	0.0647 (5)	
H5A	−1.0149	0.3590	−0.1273	0.078*	
C6A	−1.2187 (3)	0.4222 (2)	−0.09410 (14)	0.0840 (7)	
H6A	−1.2740	0.3594	−0.0848	0.101*	
C7A	−1.2915 (3)	0.5161 (3)	−0.08276 (16)	0.0940 (9)	
H7A	−1.3951	0.5146	−0.0660	0.113*	
C8A	−1.2142 (3)	0.6116 (3)	−0.09567 (15)	0.0828 (7)	
H8A	−1.2638	0.6739	−0.0882	0.099*	
C9A	−1.0608 (2)	0.61061 (17)	−0.11997 (11)	0.0585 (5)	
C10A	−0.9459 (2)	0.69870 (15)	−0.13613 (11)	0.0569 (4)	
N1A	−0.80682 (17)	0.65359 (11)	−0.15608 (9)	0.0493 (3)	
O1A	−0.4285 (3)	0.85939 (17)	−0.06654 (12)	0.1289 (9)	
O2A	−0.55221 (17)	0.74356 (11)	−0.00883 (8)	0.0626 (4)	
O3A	−0.71648 (17)	0.48833 (10)	−0.16856 (10)	0.0637 (3)	
O4A	−0.9621 (2)	0.79203 (12)	−0.13410 (10)	0.0829 (5)	
C1B	0.40479 (19)	1.30491 (12)	0.57084 (10)	0.0425 (3)	
C2B	0.5016 (2)	1.37468 (13)	0.66499 (10)	0.0454 (4)	
H2B1	0.4301	1.4222	0.6920	0.054*	
H2B2	0.5286	1.3267	0.7015	0.054*	
C3B	0.67039 (19)	1.55202 (12)	0.66280 (10)	0.0405 (3)	
C4B	0.83781 (19)	1.58248 (12)	0.64317 (10)	0.0395 (3)	
C5B	0.9136 (2)	1.67982 (13)	0.63322 (11)	0.0470 (4)	
H5B	0.8603	1.7406	0.6396	0.056*	
C6B	1.0719 (2)	1.68341 (15)	0.61333 (12)	0.0538 (4)	
H6B	1.1261	1.7478	0.6059	0.065*	
C7B	1.1523 (2)	1.59280 (16)	0.60421 (11)	0.0544 (4)	
H7B	1.2594	1.5979	0.5913	0.065*	
C8B	1.0751 (2)	1.49488 (14)	0.61414 (11)	0.0492 (4)	
H8B	1.1285	1.4342	0.6082	0.059*	
C9B	0.91670 (19)	1.49099 (12)	0.63309 (9)	0.0401 (3)	
C10B	0.7997 (2)	1.39932 (12)	0.64460 (10)	0.0422 (3)	
N1B	0.65796 (16)	1.44176 (10)	0.66338 (9)	0.0422 (3)	
O1B	0.45570 (17)	1.30518 (12)	0.50517 (8)	0.0671 (4)	
O2B	0.26246 (15)	1.24440 (9)	0.57228 (8)	0.0516 (3)	
O3B	0.56253 (15)	1.60611 (10)	0.67685 (9)	0.0556 (3)	
O4B	0.81814 (16)	1.30561 (9)	0.63963 (8)	0.0569 (3)	
H1OA	−0.464 (3)	0.784 (2)	0.0486 (18)	0.101 (8)*	
H1OB	0.210 (3)	1.1927 (18)	0.5169 (16)	0.078 (7)*	
H1N2	0.126 (2)	0.8633 (16)	0.4029 (13)	0.056 (5)*	

### Atomic displacement parameters (Å^2^)

**Table:**

	*U*^11^	*U*^22^	*U*^33^	*U*^12^	*U*^13^	*U*^23^
C1	0.0628 (11)	0.0486 (9)	0.0472 (9)	0.0083 (8)	−0.0060 (8)	0.0194 (7)
C2	0.0578 (10)	0.0374 (8)	0.0453 (9)	0.0048 (7)	−0.0030 (7)	0.0135 (7)
C3	0.0358 (7)	0.0373 (7)	0.0347 (7)	0.0062 (6)	0.0053 (6)	0.0102 (6)
C4	0.0475 (9)	0.0371 (7)	0.0457 (8)	0.0063 (6)	−0.0004 (7)	0.0135 (6)
C5	0.0539 (9)	0.0381 (8)	0.0491 (9)	0.0017 (7)	−0.0016 (7)	0.0077 (7)
C6	0.0367 (7)	0.0350 (7)	0.0358 (7)	0.0037 (6)	0.0059 (6)	0.0098 (6)
C7	0.0418 (8)	0.0365 (7)	0.0397 (8)	0.0067 (6)	0.0058 (6)	0.0110 (6)
C8	0.0379 (7)	0.0409 (8)	0.0365 (7)	0.0083 (6)	0.0067 (6)	0.0129 (6)
C9	0.0550 (9)	0.0395 (8)	0.0492 (9)	0.0098 (7)	−0.0045 (7)	0.0169 (7)
C10	0.0605 (10)	0.0387 (8)	0.0508 (9)	0.0060 (7)	−0.0050 (8)	0.0112 (7)
C11	0.0446 (8)	0.0473 (8)	0.0365 (8)	0.0084 (7)	0.0030 (6)	0.0117 (6)
C12	0.0550 (9)	0.0477 (9)	0.0421 (8)	0.0130 (7)	0.0021 (7)	0.0207 (7)
C13	0.0522 (9)	0.0382 (8)	0.0437 (8)	0.0076 (7)	0.0047 (7)	0.0138 (6)
C14	0.0744 (13)	0.0714 (12)	0.0439 (10)	0.0109 (10)	−0.0080 (9)	0.0223 (9)
N1	0.0493 (8)	0.0489 (8)	0.0396 (7)	0.0038 (6)	−0.0030 (6)	0.0103 (6)
N2	0.0432 (7)	0.0340 (6)	0.0364 (6)	0.0068 (5)	−0.0026 (5)	0.0063 (5)
N3	0.0396 (6)	0.0400 (6)	0.0358 (6)	0.0054 (5)	−0.0006 (5)	0.0088 (5)
O1	0.0505 (6)	0.0348 (5)	0.0468 (6)	0.0030 (4)	−0.0050 (5)	0.0100 (5)
O2	0.0673 (8)	0.0539 (7)	0.0438 (6)	0.0057 (6)	−0.0126 (5)	0.0126 (5)
C1A	0.0593 (10)	0.0482 (9)	0.0460 (9)	−0.0015 (8)	0.0067 (8)	0.0126 (7)
C2A	0.0667 (11)	0.0522 (9)	0.0428 (9)	0.0047 (8)	0.0062 (8)	0.0147 (7)
C3A	0.0489 (9)	0.0448 (8)	0.0397 (8)	0.0092 (7)	−0.0003 (7)	0.0055 (7)
C4A	0.0451 (9)	0.0580 (10)	0.0373 (8)	0.0054 (7)	−0.0017 (7)	0.0034 (7)
C5A	0.0617 (11)	0.0676 (12)	0.0490 (10)	−0.0037 (9)	0.0045 (8)	0.0058 (9)
C6A	0.0640 (13)	0.1115 (19)	0.0524 (12)	−0.0225 (13)	0.0051 (10)	0.0102 (12)
C7A	0.0479 (12)	0.154 (3)	0.0619 (14)	0.0113 (15)	0.0099 (10)	0.0095 (15)
C8A	0.0557 (12)	0.121 (2)	0.0680 (14)	0.0321 (13)	0.0120 (10)	0.0173 (13)
C9A	0.0498 (10)	0.0750 (12)	0.0404 (9)	0.0219 (9)	−0.0015 (7)	0.0044 (8)
C10A	0.0659 (11)	0.0573 (10)	0.0384 (8)	0.0239 (9)	−0.0017 (8)	0.0038 (7)
N1A	0.0500 (8)	0.0448 (7)	0.0424 (7)	0.0095 (6)	−0.0027 (6)	0.0053 (6)
O1A	0.1529 (18)	0.1220 (15)	0.0688 (10)	−0.0829 (14)	−0.0089 (11)	0.0354 (10)
O2A	0.0641 (8)	0.0649 (8)	0.0428 (7)	−0.0146 (6)	−0.0080 (6)	0.0175 (6)
O3A	0.0600 (8)	0.0566 (7)	0.0794 (9)	0.0226 (6)	0.0205 (7)	0.0204 (7)
O4A	0.1137 (13)	0.0643 (9)	0.0734 (10)	0.0459 (9)	0.0155 (9)	0.0164 (7)
C1B	0.0432 (8)	0.0369 (7)	0.0429 (8)	0.0079 (6)	0.0024 (6)	0.0103 (6)
C2B	0.0483 (9)	0.0400 (8)	0.0426 (8)	0.0018 (6)	0.0056 (7)	0.0108 (6)
C3B	0.0431 (8)	0.0367 (7)	0.0374 (7)	0.0087 (6)	0.0009 (6)	0.0096 (6)
C4B	0.0410 (8)	0.0365 (7)	0.0358 (7)	0.0082 (6)	0.0000 (6)	0.0085 (6)
C5B	0.0493 (9)	0.0408 (8)	0.0484 (9)	0.0073 (7)	0.0037 (7)	0.0155 (7)
C6B	0.0514 (9)	0.0533 (9)	0.0506 (9)	−0.0018 (7)	0.0043 (7)	0.0170 (8)
C7B	0.0390 (8)	0.0720 (11)	0.0448 (9)	0.0057 (8)	0.0031 (7)	0.0130 (8)
C8B	0.0434 (8)	0.0551 (9)	0.0424 (8)	0.0167 (7)	0.0002 (7)	0.0076 (7)
C9B	0.0411 (8)	0.0378 (7)	0.0333 (7)	0.0088 (6)	−0.0035 (6)	0.0057 (6)
C10B	0.0465 (8)	0.0359 (7)	0.0356 (7)	0.0095 (6)	−0.0036 (6)	0.0056 (6)
N1B	0.0421 (7)	0.0339 (6)	0.0445 (7)	0.0049 (5)	0.0015 (5)	0.0092 (5)
O1B	0.0629 (8)	0.0824 (9)	0.0427 (7)	−0.0045 (7)	0.0081 (6)	0.0092 (6)
O2B	0.0517 (7)	0.0436 (6)	0.0470 (6)	−0.0037 (5)	0.0027 (5)	0.0058 (5)
O3B	0.0522 (7)	0.0508 (7)	0.0704 (8)	0.0220 (5)	0.0183 (6)	0.0208 (6)
O4B	0.0673 (8)	0.0347 (6)	0.0633 (7)	0.0158 (5)	0.0027 (6)	0.0124 (5)

### Geometric parameters (Å, º)

**Table:**

C1—N1	1.332 (2)	C3A—N1A	1.387 (2)
C1—C2	1.380 (2)	C3A—C4A	1.486 (2)
C1—H1	0.9300	C4A—C5A	1.377 (3)
C2—C3	1.384 (2)	C4A—C9A	1.391 (3)
C2—H2	0.9300	C5A—C6A	1.388 (3)
C3—C4	1.384 (2)	C5A—H5A	0.9300
C3—C6	1.5058 (19)	C6A—C7A	1.392 (4)
C4—C5	1.378 (2)	C6A—H6A	0.9300
C4—H4	0.9300	C7A—C8A	1.384 (4)
C5—N1	1.329 (2)	C7A—H7A	0.9300
C5—H5	0.9300	C8A—C9A	1.375 (3)
C6—O1	1.2312 (17)	C8A—H8A	0.9300
C6—N2	1.3376 (19)	C9A—C10A	1.477 (3)
C7—N3	1.2775 (19)	C10A—O4A	1.211 (2)
C7—C8	1.456 (2)	C10A—N1A	1.386 (2)
C7—H7	0.9300	O2A—H1OA	1.00 (3)
C8—C13	1.388 (2)	C1B—O1B	1.197 (2)
C8—C9	1.392 (2)	C1B—O2B	1.3123 (19)
C9—C10	1.368 (2)	C1B—C2B	1.517 (2)
C9—H9	0.9300	C2B—N1B	1.445 (2)
C10—C11	1.390 (2)	C2B—H2B1	0.9700
C10—H10	0.9300	C2B—H2B2	0.9700
C11—O2	1.3619 (18)	C3B—O3B	1.2036 (18)
C11—C12	1.381 (2)	C3B—N1B	1.4038 (18)
C12—C13	1.389 (2)	C3B—C4B	1.480 (2)
C12—H12	0.9300	C4B—C5B	1.378 (2)
C13—H13	0.9300	C4B—C9B	1.394 (2)
C14—O2	1.425 (2)	C5B—C6B	1.381 (2)
C14—H14A	0.9600	C5B—H5B	0.9300
C14—H14B	0.9600	C6B—C7B	1.391 (3)
C14—H14C	0.9600	C6B—H6B	0.9300
N2—N3	1.3811 (16)	C7B—C8B	1.388 (2)
N2—H1N2	0.87 (2)	C7B—H7B	0.9300
C1A—O1A	1.180 (2)	C8B—C9B	1.375 (2)
C1A—O2A	1.284 (2)	C8B—H8B	0.9300
C1A—C2A	1.516 (2)	C9B—C10B	1.484 (2)
C2A—N1A	1.446 (2)	C10B—O4B	1.2114 (18)
C2A—H2A1	0.9700	C10B—N1B	1.386 (2)
C2A—H2A2	0.9700	O2B—H1OB	0.93 (2)
C3A—O3A	1.2090 (19)		
			
N1—C1—C2	122.73 (15)	C5A—C4A—C9A	122.03 (18)
N1—C1—H1	118.6	C5A—C4A—C3A	130.83 (17)
C2—C1—H1	118.6	C9A—C4A—C3A	107.11 (16)
C1—C2—C3	119.45 (15)	C4A—C5A—C6A	117.2 (2)
C1—C2—H2	120.3	C4A—C5A—H5A	121.4
C3—C2—H2	120.3	C6A—C5A—H5A	121.4
C4—C3—C2	117.70 (14)	C5A—C6A—C7A	120.7 (2)
C4—C3—C6	124.47 (13)	C5A—C6A—H6A	119.7
C2—C3—C6	117.81 (13)	C7A—C6A—H6A	119.7
C5—C4—C3	119.02 (14)	C8A—C7A—C6A	121.9 (2)
C5—C4—H4	120.5	C8A—C7A—H7A	119.1
C3—C4—H4	120.5	C6A—C7A—H7A	119.1
N1—C5—C4	123.38 (15)	C9A—C8A—C7A	117.3 (2)
N1—C5—H5	118.3	C9A—C8A—H8A	121.4
C4—C5—H5	118.3	C7A—C8A—H8A	121.4
O1—C6—N2	123.53 (13)	C8A—C9A—C4A	121.0 (2)
O1—C6—C3	119.79 (13)	C8A—C9A—C10A	130.3 (2)
N2—C6—C3	116.68 (12)	C4A—C9A—C10A	108.63 (16)
N3—C7—C8	120.32 (13)	O4A—C10A—N1A	124.2 (2)
N3—C7—H7	119.8	O4A—C10A—C9A	129.96 (19)
C8—C7—H7	119.8	N1A—C10A—C9A	105.85 (15)
C13—C8—C9	118.05 (14)	C10A—N1A—C3A	112.07 (15)
C13—C8—C7	121.51 (14)	C10A—N1A—C2A	122.47 (15)
C9—C8—C7	120.44 (13)	C3A—N1A—C2A	124.74 (14)
C10—C9—C8	121.12 (14)	C1A—O2A—H1OA	112.8 (14)
C10—C9—H9	119.4	O1B—C1B—O2B	125.65 (15)
C8—C9—H9	119.4	O1B—C1B—C2B	123.35 (15)
C9—C10—C11	120.42 (15)	O2B—C1B—C2B	110.99 (14)
C9—C10—H10	119.8	N1B—C2B—C1B	111.00 (13)
C11—C10—H10	119.8	N1B—C2B—H2B1	109.4
O2—C11—C12	125.81 (14)	C1B—C2B—H2B1	109.4
O2—C11—C10	114.62 (14)	N1B—C2B—H2B2	109.4
C12—C11—C10	119.54 (14)	C1B—C2B—H2B2	109.4
C11—C12—C13	119.55 (14)	H2B1—C2B—H2B2	108.0
C11—C12—H12	120.2	O3B—C3B—N1B	124.21 (15)
C13—C12—H12	120.2	O3B—C3B—C4B	130.12 (14)
C8—C13—C12	121.27 (14)	N1B—C3B—C4B	105.68 (12)
C8—C13—H13	119.4	C5B—C4B—C9B	121.58 (15)
C12—C13—H13	119.4	C5B—C4B—C3B	130.30 (14)
O2—C14—H14A	109.5	C9B—C4B—C3B	108.11 (13)
O2—C14—H14B	109.5	C4B—C5B—C6B	117.23 (15)
H14A—C14—H14B	109.5	C4B—C5B—H5B	121.4
O2—C14—H14C	109.5	C6B—C5B—H5B	121.4
H14A—C14—H14C	109.5	C5B—C6B—C7B	121.48 (16)
H14B—C14—H14C	109.5	C5B—C6B—H6B	119.3
C5—N1—C1	117.72 (14)	C7B—C6B—H6B	119.3
C6—N2—N3	118.70 (12)	C8B—C7B—C6B	121.01 (16)
C6—N2—H1N2	121.2 (13)	C8B—C7B—H7B	119.5
N3—N2—H1N2	119.8 (12)	C6B—C7B—H7B	119.5
C7—N3—N2	116.20 (12)	C9B—C8B—C7B	117.49 (15)
C11—O2—C14	117.57 (14)	C9B—C8B—H8B	121.3
O1A—C1A—O2A	124.36 (17)	C7B—C8B—H8B	121.3
O1A—C1A—C2A	120.76 (17)	C8B—C9B—C4B	121.20 (15)
O2A—C1A—C2A	114.78 (15)	C8B—C9B—C10B	130.71 (14)
N1A—C2A—C1A	113.27 (14)	C4B—C9B—C10B	108.08 (13)
N1A—C2A—H2A1	108.9	O4B—C10B—N1B	124.78 (15)
C1A—C2A—H2A1	108.9	O4B—C10B—C9B	129.25 (15)
N1A—C2A—H2A2	108.9	N1B—C10B—C9B	105.98 (12)
C1A—C2A—H2A2	108.9	C10B—N1B—C3B	112.14 (13)
H2A1—C2A—H2A2	107.7	C10B—N1B—C2B	123.63 (13)
O3A—C3A—N1A	124.53 (16)	C3B—N1B—C2B	123.10 (13)
O3A—C3A—C4A	129.13 (16)	C1B—O2B—H1OB	112.4 (14)
N1A—C3A—C4A	106.33 (14)		
			
N1—C1—C2—C3	0.0 (3)	C3A—C4A—C9A—C10A	0.63 (18)
C1—C2—C3—C4	0.4 (2)	C8A—C9A—C10A—O4A	−2.3 (3)
C1—C2—C3—C6	−178.24 (15)	C4A—C9A—C10A—O4A	179.28 (18)
C2—C3—C4—C5	−0.5 (2)	C8A—C9A—C10A—N1A	178.19 (19)
C6—C3—C4—C5	178.00 (15)	C4A—C9A—C10A—N1A	−0.28 (18)
C3—C4—C5—N1	0.3 (3)	O4A—C10A—N1A—C3A	−179.81 (16)
C4—C3—C6—O1	−167.66 (15)	C9A—C10A—N1A—C3A	−0.21 (18)
C2—C3—C6—O1	10.8 (2)	O4A—C10A—N1A—C2A	9.5 (3)
C4—C3—C6—N2	11.7 (2)	C9A—C10A—N1A—C2A	−170.92 (14)
C2—C3—C6—N2	−169.80 (14)	O3A—C3A—N1A—C10A	−179.69 (16)
N3—C7—C8—C13	170.94 (14)	C4A—C3A—N1A—C10A	0.59 (17)
N3—C7—C8—C9	−9.4 (2)	O3A—C3A—N1A—C2A	−9.2 (3)
C13—C8—C9—C10	0.1 (3)	C4A—C3A—N1A—C2A	171.05 (14)
C7—C8—C9—C10	−179.63 (16)	C1A—C2A—N1A—C10A	80.2 (2)
C8—C9—C10—C11	−1.8 (3)	C1A—C2A—N1A—C3A	−89.3 (2)
C9—C10—C11—O2	179.91 (16)	O1B—C1B—C2B—N1B	1.4 (2)
C9—C10—C11—C12	1.8 (3)	O2B—C1B—C2B—N1B	−179.70 (12)
O2—C11—C12—C13	−178.07 (16)	O3B—C3B—C4B—C5B	−2.0 (3)
C10—C11—C12—C13	−0.2 (3)	N1B—C3B—C4B—C5B	178.67 (15)
C9—C8—C13—C12	1.5 (2)	O3B—C3B—C4B—C9B	179.29 (16)
C7—C8—C13—C12	−178.76 (15)	N1B—C3B—C4B—C9B	−0.02 (16)
C11—C12—C13—C8	−1.5 (3)	C9B—C4B—C5B—C6B	−0.4 (2)
C4—C5—N1—C1	0.0 (3)	C3B—C4B—C5B—C6B	−178.94 (15)
C2—C1—N1—C5	−0.1 (3)	C4B—C5B—C6B—C7B	−0.4 (2)
O1—C6—N2—N3	−1.0 (2)	C5B—C6B—C7B—C8B	0.5 (3)
C3—C6—N2—N3	179.68 (12)	C6B—C7B—C8B—C9B	0.1 (2)
C8—C7—N3—N2	179.59 (13)	C7B—C8B—C9B—C4B	−0.9 (2)
C6—N2—N3—C7	−179.03 (13)	C7B—C8B—C9B—C10B	177.81 (15)
C12—C11—O2—C14	−8.0 (3)	C5B—C4B—C9B—C8B	1.0 (2)
C10—C11—O2—C14	174.13 (16)	C3B—C4B—C9B—C8B	179.88 (13)
O1A—C1A—C2A—N1A	−161.2 (2)	C5B—C4B—C9B—C10B	−177.90 (13)
O2A—C1A—C2A—N1A	22.3 (2)	C3B—C4B—C9B—C10B	0.93 (16)
O3A—C3A—C4A—C5A	1.4 (3)	C8B—C9B—C10B—O4B	−0.1 (3)
N1A—C3A—C4A—C5A	−178.94 (17)	C4B—C9B—C10B—O4B	178.70 (15)
O3A—C3A—C4A—C9A	179.55 (17)	C8B—C9B—C10B—N1B	179.68 (15)
N1A—C3A—C4A—C9A	−0.75 (17)	C4B—C9B—C10B—N1B	−1.50 (16)
C9A—C4A—C5A—C6A	−0.7 (3)	O4B—C10B—N1B—C3B	−178.65 (14)
C3A—C4A—C5A—C6A	177.28 (17)	C9B—C10B—N1B—C3B	1.54 (16)
C4A—C5A—C6A—C7A	0.5 (3)	O4B—C10B—N1B—C2B	−10.5 (2)
C5A—C6A—C7A—C8A	0.0 (3)	C9B—C10B—N1B—C2B	169.65 (13)
C6A—C7A—C8A—C9A	−0.3 (3)	O3B—C3B—N1B—C10B	179.65 (14)
C7A—C8A—C9A—C4A	0.1 (3)	C4B—C3B—N1B—C10B	−0.99 (16)
C7A—C8A—C9A—C10A	−178.19 (19)	O3B—C3B—N1B—C2B	11.5 (2)
C5A—C4A—C9A—C8A	0.4 (3)	C4B—C3B—N1B—C2B	−169.17 (13)
C3A—C4A—C9A—C8A	−178.01 (17)	C1B—C2B—N1B—C10B	−71.42 (18)
C5A—C4A—C9A—C10A	179.01 (15)	C1B—C2B—N1B—C3B	95.41 (16)

### Hydrogen-bond geometry (Å, º)

**Table:**

*D*—H···*A*	*D*—H	H···*A*	*D*···*A*	*D*—H···*A*
O2*A*—H1*OA*···N1	1.00 (3)	1.60 (3)	2.5997 (19)	177 (2)
O2*B*—H1*OB*···O1	0.93 (2)	1.75 (2)	2.6736 (16)	170 (2)
N2—H1*N*2···O4*B*^i^	0.87 (2)	2.22 (2)	3.0549 (18)	161 (2)
C2*A*—H2*A*2···O3*B*^ii^	0.97	2.57	3.477 (2)	156
C5*B*—H5*B*···O1^iii^	0.93	2.51	3.158 (2)	126
C7*B*—H7*B*···O3*B*^iv^	0.93	2.55	3.275 (2)	135
C5—H5···O3*A*^v^	0.93	2.48	3.341 (2)	154

Symmetry codes: (i) −*x*+1, −*y*+2, −*z*+1; (ii) *x*−1, *y*−1, *z*−1; (iii) −*x*+1, −*y*+3, −*z*+1; (iv) *x*+1, *y*, *z*; (v) −*x*−1, −*y*+1, −*z*.
